# Supplementary material for: Optimized Therapeutic 177Lu-Labeled PSMA-Targeted Ligands with Improved Pharmacokinetic Characteristics for Prostate Cancer
Source: Pharmaceuticals (Basel). 2022 Dec 9;15(12):1530. doi: 10.3390/ph15121530 (PMC9782218; doi:10.3390/ph15121530)
Supplement: Supplementary file 1 [file pharmaceuticals-15-01530-s001.zip › pharmaceuticals-2023634-Supplementary Material.pdf]

Supplementary Material of

Optimized Therapeutic ^{177}Lu-labeled PSMA-targeted Radiopharmaceuticals with Improved Pharmacokinetic Characteristics for Prostate Cancer

Yitian Wu[1], Xiaojun Zhang[1], Xiaojiang Duan[2], Xing Yang[2, 3], Feng Wang [4, *] and Jinming Zhang[1, *]

1 Department of Nuclear Medicine, Chinese PLA General Hospital, Beijing 100853, China

2 Department of Nuclear Medicine, Peking University First Hospital, Beijing 100034, China

3 Institute of Medical Technology, Peking University, Beijing 100191, China

4 Department of Nuclear Medicine, Nanjing First Hospital, Nanjing Medical University, Nanjing 210006, China

* Correspondence: Feng Wang: fengwangcn@hotmail.com and Jinming Zhang: zhangjm301@163.com

Chemical synthesis of PSMA-Q, PSMA-4PY and PSMA-BP

All the ligands were synthesized via solid-phase synthesis which was performed according to Fmoc peptide synthesis protocol.

i) Fmoc-Glu-urea-Lys-resin (0.22 mmol/g, 100 mg) was added into the solid phase synthesis tube, the compound was rinsed with DCM (3 × 5 min × 2 mL) and DMF (3 × 5 min × 2 mL). To remove Fmoc, the compound was rinsed with DMF containing 20% piperidine (1 × 2 min × 2 mL, 2 × 10 min × 2 mL) and DMF (6 × 1 min × 2 mL).

ii) A mixture of Fmoc-3-(3-quinoline)-D-alanine (Q) or Fmoc-3-(4-pyridyl)-D-alanine (4PY) or Fmoc-3-(biphenyl)-D-alanine (BP) (0.06 mmol, 26.2 mg), HBTU (0.072 mmol, 27 mg), HOBt (0.072 mmol, 10 mg), DIPEA (0.15 mmol, 25 μL) was stirred in DMF (3 mL) at room temperature for 15 min. The activated 2-pyridine-alanine was added into the cleaned resin and reacted under nitrogen for 1 hour and then rinsed with DMF (6 × 1 min × 2 mL). To remove Fmoc, the compound was rinsed with DMF containing 20% piperidine (1 × 2 min × 2 mL, 2 × 10 min × 2 mL) and DMF (6 × 1 min × 2 mL).

iii) A mixture of trans-4-(Fmoc-aminomethyl)-cyclohexanecarboxylic acid (3 M, 0.06 mmol, 23 mg), HBTU (0.072 mmol, 27 mg), HOBt (0.072 mmol, 10 mg), DIPEA (0.15 mmol, 25 μL) was stirred in 3 mL DMF at room temperature for 15 min. The activated cyclohexane formic acid was added into the cleaned resin and reacted under nitrogen for 1 hour and then rinsed with DMF (6 × 1 min × 2 mL). To remove Fmoc, the compound was rinsed with DMF containing 20% piperidine (1 × 2 min × 2 mL, 2 × 10 min × 2 mL) and DMF (6 × 1 min × 2 mL).

iiii) A mixture of DOTA tert-butyl ester (3 M, 0.06 mmol, 34.4 mg), HBTU (0.072 mmol, 27 mg), HOBt (0.072 mmol, 10 mg) and DIPEA (0.15 mmol, 25 μL) was stirred in 3 mL of DMF at room temperature for 15 min. The activated DOTA tert-butyl ester was added to the cleaned resin and reacted under nitrogen for 1 hour and then rinsed with DMF (6 × 1 min × 2 mL). A solution of TFA (4.5 mL), TIPS (250 μL), H_2O (250 μL) was added and reacted at room temperature for 2 h. The filtrate was collected and washed with TFA (2 mL). The final products (PSMA-Q, PSMA-4PY and PSMA-BP) were purified by HPLC.

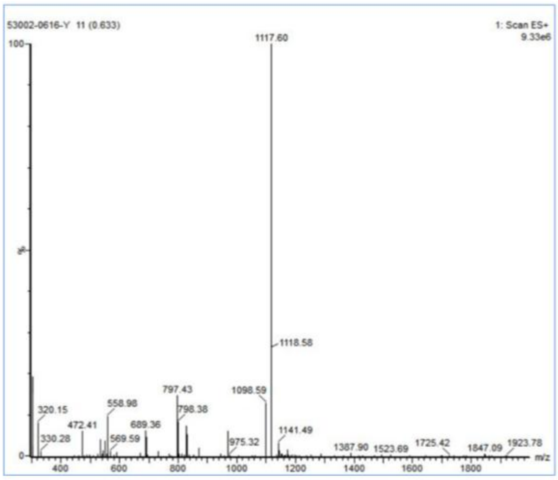

PSMA-4PY

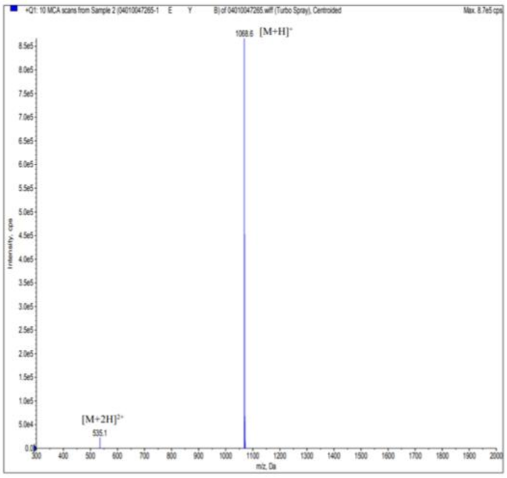

PSMA-BP

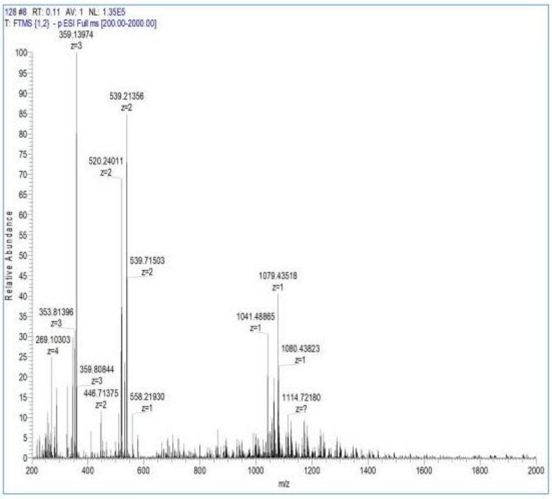

PSMA-Q

Figure S1. ESI-MS chromatogram of novel PSMA ligands
